# Spark Analysis Based on the CNN-GRU Model for WEDM Process

**DOI:** 10.3390/mi12060702

**Published:** 2021-06-16

**Authors:** Changhong Liu, Xingxin Yang, Shaohu Peng, Yongjun Zhang, Lingxi Peng, Ray Y. Zhong

**Affiliations:** 1 School of Electromechanical Engineering, Guangdong University of Technology, Guangzhou 510006, China; lch@gzhu.edu.cn; 2 School of Mechanical and Electrical Engineering, Guangzhou University, Guangzhou 510006, China; flyingday@139.com; 3 School of Electronics and Communication Engineering, Guangzhou University, Guangzhou 510006, China; gdnhyxx@163.com (X.Y.); pengsh@gzhu.edu.cn (S.P.); 4 Department of Industrial and Manufacturing Systems Engineering, The University of Hong Kong, Hong Kong 999077, China; zhongzry@hku.hk

**Keywords:** wire electrical discharge machining (WEDM), deep learning, spark analysis, convolution neural network (CNN), gated recurrent unit (GRU)

## Abstract

Wire electrical discharge machining (WEDM), widely used to fabricate micro and precision parts in manufacturing industry, is a nontraditional machining method using discharge energy which is transformed into thermal energy to efficiently remove materials. A great amount of research has been conducted based on pulse characteristics. However, the spark image-based approach has little research reported. This paper proposes a discharge spark image-based approach. A model is introduced to predict the discharge status using spark image features through a synchronous high-speed image and waveform acquisition system. First, the relationship between the spark image features (e.g., area, energy, energy density, distribution, etc.) and discharge status is explored by a set of experiments). Traditional methods have claimed that pulse waveform of “short” status is related to the status of non-machining while through our research, it is concluded that this is not always true by conducting experiments based on the spark images. Second, a deep learning model based on Convolution neural network (CNN) and Gated recurrent unit (GRU) is proposed to predict the discharge status. A time series of spark image features extracted by CNN form a 3D feature space is used to predict the discharge status through GRU. Moreover, a quantitative labeling method of machining state is proposed to improve the stability of the model. Due the effective features and the quantitative labeling method, the proposed approach achieves better predict result comparing with the single GRU model.

## 1. Introduction

Wire electrical discharge machining (WEDM) is a non-conventional machining method used to remove material through the high temperature produced by a series of repetitive electrical discharge of small duration and huge current density between the wire tool and work piece [1,2,3,4]. Due to the minute amount of spark erosion, WEDM is usually used in the machining of micro and precision parks. For example, Ahmed et al. [5] conducted the experiment on the manufacture of high-aspect-ratio thin structures of micrometer thickness (117–500 μm) from D2 steel through WEDM. In order to produce microchannels with desired/target geometry and acceptable surface quality, Saleh et al. [6] carried out the results of an investigation on the capacity of WEDM to produce microchannels in the nickel-based alloy, Monel 400. WEDM is one of typical kind of EDM, they are developed by using the phenomenon of spark erosion, and a lot of research has been carried out in various aspects relating to improving performance measures, optimizing the process variables, and monitoring and controlling the sparking process [4,7].

Since the advent of EDM, a great deal of research had been focused on its mechanism from the perspective of energy. In essence, EDM is a process of energy conversion which mainly turns electric energy into thermal energy. The conservation of energy and charge is the basis of analyzing spark discharge phenomenon [8]. Ablyaz et al. [9] developed the mathematical modeling of quality parameters of EDM cut surfaces based the physics of EDM process, i.e., the transformation of electrical energy of spark discharge between the tool and the workpieces into thermal energy resulting in removing the material. Through reviewing previous research, Shabgard et al. [3] demonstrated that the fraction of heat going into the electrodes is a function of input parameters of the process. Based on pulse classification and a thermal model, Dekeyser et al. [10] designed an expert system for WEDM to improve the level of machine autonomy. By using a simple empirical concept, Gostimirovic et al. [11] found that the thermal state defined in the discharge zone was directly influenced by the discharge current and pulse duration as well it determined the machining characteristics of EDM predominantly. To predict the shape of crater, MRR and TWR, a two-dimensional axi-symmetric numerical finite element method (FEM) model of single spark EDM process had been investigated based on more realistic assumptions such as Gaussian distribution of heat flux, time and energy dependent spark radius, etc. [12]. In order to simulate and analysis the crater for various plasma flushing efficiency during wire electrical discharge turning (WEDT), FEM was proposed by Giridharan et al. [13] and the model predicted erosion energy to form a crater with an average absolute error of 17.86% which was still not precise enough. On one hand, the model based on mathematics and physics induced large deviations from actual practice because of inevitable assumptions made in the physical modeling of the process. On the other hand, the EDM process was highly complex and stochastic in nature, and involved many subjects such as electric, magnetic, thermal, dynamic, etc. Consequently, it was quite difficult to model the EDM process due to non-linear relationship between input process and output performance parameters.

EDM aims to achieve higher machining productivity with a desired accuracy and surface finish. Therefore, the present problem must be considered to be a multi-objective optimization problem [14]. In fact, many studies about process performance can be categorized as modeling and optimization. During the process of machining superelastic shape memory nitinol (Ni5.8Ti), Chaudhari et al. [15] used a heat-transfer search algorithm to efficiently predict and optimize the WEDM process parameters. Yuan et al. [16] developed an intelligent integrated architecture based on Gaussian process regression (GPR) models, multi-objective genetic algorithm (MOGA) and clustering for the WEDM-HS process optimization. A drawback of GPR method is that optimization iterative process is a nonlinear problem which may cause the difficulties of model convergence. An ANN model can be applied to replace the complex mathematical approximation of the relationship between input process parameters and output response during WEDM process [17,18]. The first model of the ANN was given by McCulloch and Pitts, and the ANN model was preliminary used to predict the process performance in the WEDM process [19]. So far, ANN techniques and heuristic algorithms have been used to model and optimize process parameter settings of EDM in a lot of studies [20,21,22]. Sidhu et al. [23] used ANN to predict residual stress during EDM of Al/SiC metal matrix composites after finding out the significant factors by the analysis of variance(ANOVA) method. Upadhyay et al. [24] attempted to directly use ANN model to find the significant factors which impacted the MRR of the micro-EDM process with additives in the dielectric fluid. Sagbas et al. [25] combined Taugchi method and BPNN model to effectively help engineers to determine the optimum process parameters during EDM. In the investigation of MRR and SR in WEDM process for cementation alloy steel, Shakeri et al. [26] also formulated comparison of experimental tests with regression and ANN models in order to determine the settings of pulse current, frequency of pulse, wire speed, and servo speed for estimation of MRR and SR. Based on their results, BPNN yielded better prediction. ANFIS combines fuzzy logic and neural networks organically and makes a fuzzy system more systematic and less dependent upon expert knowledge. Also, an interval type-2 fuzzy-integrated AHP-ARAS method is designed to select the best WEDM parameter settings as well compute the weightage of the criteria by applying the ARAS ranking method and AHP method, respectively [27]. Suganthi et al. [28] carried out the comparative experiments about ANN model and ANFIS model and revealed the fact that ANFIS outperformed to ANN in terms of modeling and prediction accuracy. Sarkheyli et al. [29] proposed a hybrid technique anchored in ANFIS and modified genetic algorithm (MGA) to train a model to predict the SR and MRR in WEDM process. Recently, Naresh et al. [22] also have concluded that ANFIS model gave more exact and effective soft computing method when compared to ANN model for superior prediction of WEDM process responses like MRR and SR of Nitinol alloy. In addition, Somashekhar et al. [30] combined ANN and genetic algorithm (GA) in optimizing the MRR in micro-EDM that the back-propagation network data along with the GA can successfully synthesize optimum input condition to maximize the MRR. Ong et al. [31] developed a small mean-squared error (MSE) model of radial basis function neural network to predict the MRR and EWR of the EDM process. Ming et al. [32] conducted cutting parameter optimization in the WEDM process by integrating ANN, and wolf pack algorithm based on the strategy of the leader (LWPA). It was found that the ANN-LWPA integration system has some advantages on reducing the value of fitness functions by comparison with the experimental regression model, ANN model, and conventional LWPA result. Furthermore, Yan et al. successively developed a servo control system based on fuzzy rule-based control strategy and adjusting strategy, as well a hierarchical adaptive control system based on the estimation of workpiece height on-line by using ANN to reduce the wire breakage and improve the machining stability and speed compared to the commonly used gap voltage control system [33,34].

From previous research about modeling and optimization for WEDM (or EMD), classical approaches such as Taguchi, ANN, etc. or their hybrid methods such as ANN-LWPA, GPR-MOGA, etc. are basically used, which studied performance in terms of electrical characteristics such as pulse current and wire speed. However, their research lacks a visual perspective which contains useful and important information in the WEDM process, such as images of sparks and wire vibrations.

In recent years, some novel research has emerged. Zhang et al. [35] first proposed a hybrid technique of WEDM which employs assisted ultrasonic vibration (USV) and magnetic field (MF) to improve the machine performance. Then they implemented theoretical and experimental study to illustrate its improving mechanism and gained the high MRR (44.0%) and the low SR (30.5%) performance as a result [36]. Recently, Ablyaz et al. [37] found that a 118% increase in MRR and an enhancement (613.6%) in the micro-hardness under the influence of magnetic field during the EDM process of AL-SiC metal Matrix Composite. Through analysis of the influence between pulse type and process performance indicators, it showed that MRR and TWR values increased as the number of normal pulses grew while the TWR decreased in the condition of increasing in arcs and delayed pulses [38]. Moreover, it was found that cutting rate and surface roughness were affected significantly by input parameters (Ton, Toff, SV, WF) during WEDM process of Ni-27Cu-3.15Al-2Fe-1.5Mn, and the empirical relations between them were concluded by Aggarwal et al. [39]. Gurupavan et al. [40] proposed a machine vision system which can provide wire electrode status and workpiece surface texture information in WEDM of aluminum silicon nitride (AlSi3N4) composite material via acquire the images of wire electrode and machined surface specimens using the machine vision system. Sanchez et al. [41] presented computer simulation software for the analysis of error of WEDM trapper-cutting which observably reduced experimental work. In order to address the problem about limitation of existing servo systems in machining semiconductors by WEDM, Liu et al. [42] developed a new servo system based on current pulse probability detection. Zhang et al. [43] used Wavelet moment analysis (WMA), Hu moment analysis (HMA), fractal dimension analysis (FDA), local geometric characteristics (LGC), and global geometric characteristics (GGC) to extract the waveform image features and reduce image dimension, and then based on SVM and regression, developed a two-stage classification method for discharge pulse discrimination and classification which to monitor discharge pulse on-line in WEDM-HS process. The reason is that high frequency discharge and micro-energy discharge may seriously complicate obstruction discharge signal distortion [44].

The above mentioned approaches showed good performance in some cases; however, they have limitations like low efficiency, instability, and even system breakdown [45], due to the following reasons: (1) voltage and current signals are accompanied with non-stationarity, nonlinearity, and internal coupling characteristics; (2) conventional method conducts a hysteretic control due to the discharge state changes so fast that the controlling strategy resulting from the historical state is not always suitable for the current state.

Different from the previous research, this paper presents a novel approach and perspective to predict the discharge status through spark images captured by high-speed camera. Considering the spark phenomenon in WEDM, spark images from a high-speed camera are collected and a series of experimental analyses are conducted. In the papers reviewed above, most research only focused on the relationship between processing technic and electrical parameters. However, they ignored the essential phenomenon in the process of WEMD (or EDM): the generation of electric spark. Although some research gradually begins to apply ANN [46] and other intelligent algorithms to the research of control system and on-line prediction [47,48], there is still high potential for improvement. In other words, the methods based on electrical parameters and traditional intelligent algorithms encounter a bottleneck effect due to the limitations we have mentioned above. With the increase of computing power, artificial neural network is more effective than traditional methods in image feature extraction and sequence feature extraction [49]. Recently, Zhang et al. [50] presented a novel and intelligent pulse classification method using different recurrent neural networks (RNNs) and the result verified that RNN performed well in the sequence recognition task during EDM process. Also, Lee et al. [51] combined a CNN and RNN to extract time-dependent and time-independent features during the chemical mechanical planarization process. Bustillo et al. [52] found that Adaboost ensembles provided the highest accuracy and were more easily optimized than artificial neural networks during the optimization of a friction-drilling process. In addition, Chen et al. [53] claimed that extracting signal characteristic was fairly time consuming so that they proposed a multi-scale CNN and LSTM model to apply to bearing fault diagnosis. This new study found that deep learning method performs better than traditional methods such as empirical mode decomposition, fast Fourier transform, discrete wavelet transform, etc. Considering the essential phenomena of spark during the process of WEDM and the advantages of new methods of image processing and deep learning, this paper proposes a new spark image identification method based on convolution neural network (CNN) and GRU to predict the discharge status. Through CNN, the features of spark images can be extracted by a series of convolution kernels. In order to train the deep learning network, the relationship between spark images and discharge status is achieved by mapping the voltage–current state (through their waveform areas and their power) to the spark images.

In the past studies, discrete values are basically used to define the processing state—such as open circuit, short circuit, processing, and other states. Since the discrete processing state is generally obtained through the threshold method, it is very sensitive to the boundary value. Therefore, this approach can cause unstable of the state and increase the probability of misjudgment. In order to overcome this problem, this paper proposes a continuous quantity to define the processing state—that is, the area of voltage waveform, the area of current waveform, and the continuous quantity of power are used to define the processing state. On one hand, using continuous value to evaluate machining state can greatly improve the stability of model. On the other hand, using the processing state of continuous value as the label is conducive to the design of the later neural network model, which can transform the classification task into a regression task, and avoid the problem of difficult convergence caused by the frequent jump of discrete label value under the condition of approximate characteristic input.

Since the frames in the collected spark image sequence are related to each other, a current frame may retain a part of information about its previous frame. The remaining information of a frame becomes interference when a single frame is used as the input of the network model. In view of this, this paper proposes two models named “Sequence2Sequence” and “Image2Sequence” to predict the discharge status by the spark images. Both models take information about the current and past frames as input. Under these circumstances, the information of the past frame will reflect the motion trajectory and motion state of the spark, which is important to reflect the processing state.

Therefore, the definition of continuous labeling and two kinds of network models proposed in this paper are important work for determining the law between spark image and machining state. The spark image is the most essential and direct phenomenon in the process of WEDM, and the law between spark images and the processing state is conducive to the exploration of higher precision processing technology and lower cost of multi-station real-time control system.

This paper is organized as follows. In Section 2, it introduces the working principle of WEDM, the main characteristics of spark image, the principle of RNN, CNN and the dynamic time warping (DTW) algorithm. In Section 3, it introduces the synchronous acquisition and preprocessing of voltage data, current data and image data. Section 4 is about experimental setup. In Section 5, the experimental data is analyzed statistically based on the theory in Section 2. In Section 5, the data is trained based on the two different RNN models, and the results are analyzed and discussed. Finally, a conclusion of the work is provided.

## 2. Spark Feature Analysis under WEDM

### 2.1. Spark Feature

**Figure 1:**
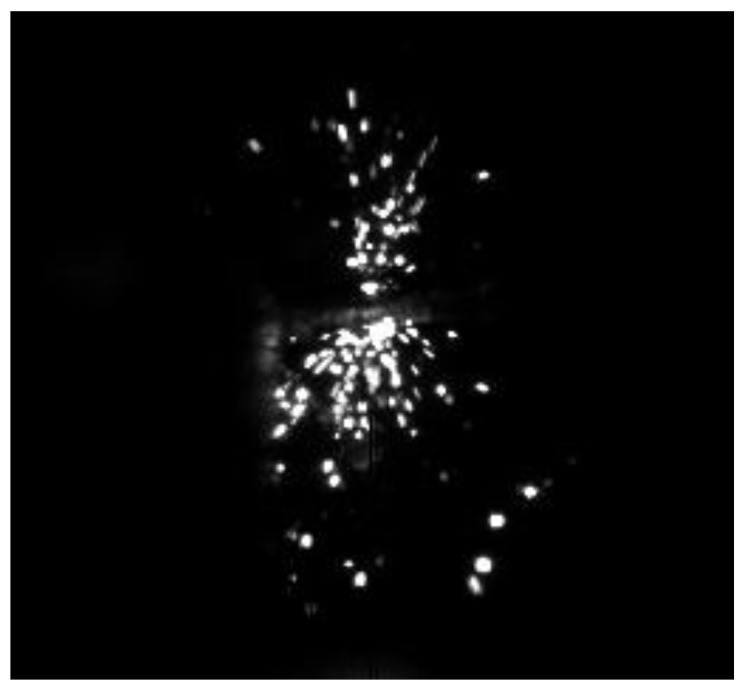
Spark image.

Figure 1 shows the image of spark during processing. Let *H*, *W* denote the height and width of the spark image, respectively. Point (*x*_0_, *y*_0_) denotes the spark center. *p*(*x*_0_, *y*_0_) denotes the pixel value of the point (*x*_0_, *y*_0_). According to the characteristics of spark, eight kinds of features were defined as follows, and the representation information is given in the Table 1.

As the working ranges of the response variables varies both in units and magnitude, normalization of data is crucial. Each response data is normalized into dimensionless values to make them comparable with each other. Various feature extraction and normalization methods are given as follows.

### 2.2. Spark Feature

Let *M*(*x*, *y*) denotes the threshold image of the spark image, then the threshold function is

(1) $$M(x,y)=\{\begin{array}{ll} 1, & P(x,y)>0\\ 0, & otherwise \end{array}$$

where *P*(*x*, *y*) denotes the pixel value of the point (*x*, *y*).

The area of spark (*S*) can be counted by the formula

(2) $$S=\sum_{x=1}^{W}\sum_{y=1}^{H}M(x,y)$$

Then the normalized area (*S_n_*) can be calculated as

(3) $$S_{n}=\frac{S}{H*W}$$

where *H*, *W* denote the height and width of the spark image, respectively.

#### 2.2.1. Energy (E)

According to the previous study [54], the Gaussian distribution of heat input proposed by Patel et al. has been used to approximate the heat from the plasma. The heat flux *q_w_(r*) at radius *r* is given by the following formula [13].

(4) $$q_{w}(r)=q_{0}e^{-4.5{\left(\frac{r}{R_{pc}}\right)}^{2}}$$

where *R_pc_* is spark radius (µm) at the work surface, and the maximum heat flux *q*_0_ can be calculated [13] as

(5) $$q_{0}=\frac{4.56F_{c}VI}{\pi R_{pc}{}^{2}}$$

where *F_c_* is the fraction of total EDM spark power going to the cathode; *V* is discharge voltage (V); *I* is discharge current (A).

Ikai et al. [55] have derived a semiempirical equation of spark radius (*R_pc_*) namely “equivalent heat input radius” as a function of discharge current (*I*) and spark on time (*T_d_*), which is more realistic as compared to other approaches. The spark radius *R_pc_* is shown as

(6) $$R_{pc}=2040I^{0.43}T_{d}{}^{0.44}$$

In this paper, the function of spark radius and energy is defined as

(7) $$E=\sum_{x=1}^{W}\sum_{y=1}^{H}P(x,y)f(d)=\sum_{x=1}^{W}\sum_{y=1}^{H}\frac{P(x,y)}{{\left(x-x_{0}\right)}^{2}+{\left(y-y_{0}\right)}^{2}}$$

where point (*x*_0_, *y*_0_) denotes the spark center of spark image, and *f*(*d*) is a function of distance (*d*) between spark point to spark center. They are calculated as

(8) $$f(d)=d^{-2}$$

(9) $$d=\sqrt{{\left(x-x_{0}\right)}^{2}+{\left(y-y_{0}\right)}^{2}}$$

Normalized energy (*E_n_*):

(10) $$K=\sum_{x^{\prime }=1}^{W}\sum_{y^{\prime }=1}^{H}255f(d)=\sum_{x^{\prime }=1}^{W}\sum_{y^{\prime }=1}^{H}\frac{255}{{\left(x^{\prime }-x_{0}\right)}^{2}+{\left(y^{\prime }-y_{0}\right)}^{2}}$$

(11) $$E_{n}=\frac{E}{K}=\sum_{x=1}^{W}\sum_{y=1}^{H}\frac{P(x,y){\left[{\left(x-x_{0}\right)}^{2}+{\left(y-y_{0}\right)}^{2}\right]}^{-1}}{\sum_{x^{\prime }=1}^{W}\sum_{y^{\prime }=1}^{H}\frac{255}{{\left(x^{\prime }-x_{0}\right)}^{2}+{\left(y^{\prime }-y_{0}\right)}^{2}}}$$

where *K* denotes the total energy when the spark image is white i.e., *P*(*x*, *y*) is 255 in Equation (7).

#### 2.2.2. Spark Energy Density (ESR)

(12) $$ESR=\frac{E_{n}}{S_{n}}$$

where *E_n_* and *S_n_* are calculated by Equations (3) and (11), respectively. Through Equation (12), it is found that *ESR* reflects the concentration of energy.

#### 2.2.3. Spark Area Distribution (SD_k_)

**Figure 2:**
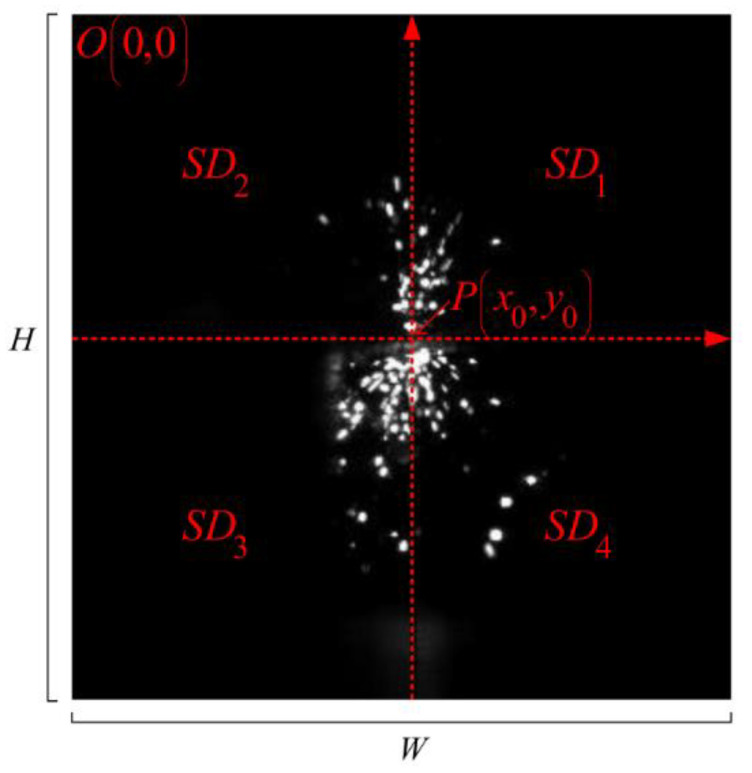
Spark area distribution (*SD_k_*).

As shown in Figure 2, the spark image is divided into four parts. According to Equation (2), the spark area of each part can be calculated as follows.

(13) $$SD_{1}=\sum_{x=x_{0}}^{W-1}\sum_{y=0}^{y_{0}-1}M(x,y)$$

(14) $$SD_{2}=\sum_{x=0}^{x_{0}-1}\sum_{y=0}^{y_{0}}M(x,y)$$

(15) $$SD_{3}=\sum_{x=0}^{x_{0}}\sum_{y=y_{0}+1}^{H-1}M(x,y)$$

(16) $$SD_{4}=\sum_{x=x_{0}+1}^{W-1}\sum_{y=y_{0}}^{H-1}M(x,y)$$

#### 2.2.4. Spark Energy Distribution (ED_k_)

Similarly to the calculations of spark area distribution, the spark energy distributions can be calculated based on Equation (7).

(17) $$ED_{1}=\sum_{x=x_{0}}^{W-1}\sum_{y=0}^{y_{0}-1}\frac{P(x,y)}{{\left(x-x_{0}\right)}^{2}+{\left(y-y_{0}\right)}^{2}}$$

(18) $$ED_{2}=\sum_{x=0}^{x_{0}-1}\sum_{y=0}^{y_{0}}\frac{P(x,y)}{{\left(x-x_{0}\right)}^{2}+{\left(y-y_{0}\right)}^{2}}$$

(19) $$ED_{3}=\sum_{x=0}^{x_{0}}\sum_{y=y_{0}+1}^{H-1}\frac{P(x,y)}{{\left(x-x_{0}\right)}^{2}+{\left(y-y_{0}\right)}^{2}}$$

(20) $$ED_{4}=\sum_{x=x_{0}+1}^{W-1}\sum_{y=y_{0}}^{H-1}\frac{P(x,y)}{{\left(x-x_{0}\right)}^{2}+{\left(y-y_{0}\right)}^{2}}$$

*ED_k_* reflects the direction of the explosion and indirectly reflects the distribution of the erosion of the workpiece.

#### 2.2.5. HU Moment

Classical geometric moments *m_pq_* of an image *I_xy_* are calculated with the equation

(21) $$m_{pq}=\sum_{x=1}^{M}\sum_{y=1}^{N}x^{p}y^{q}I_{xy}$$

Hu [56] first proposed seven invariant moments *u_1_*-*u_7_* by using the normalized central moments of second-order and third-order. HU moments are widely used to image recognition along with a series of basic properties including the rotation, translation, scale invariance [57,58].

### 2.3. Dynamic Time Warping

In the acquisition of time series data, electrical parameters and spark images are different from multiple aspects—such as sample rate, physical property, the time shift characteristics of the occurrence of phenomena, etc. Additionally, the unavoidable noise at the acquisition system also brings about time shifting between the two types of time series data even if they describe the same discharge status. Consequently, it is not appropriate to use Euclidean distance to measure the similarity of two types of time series. In every way, Euclidean distance and its variants present several drawbacks, that make inappropriate their use in certain applications [59].

(1) It compares only time series of the same length.
(2) It does not handle outliers or noise.
(3) It is very sensitive with respect to six signal transformations: shifting, uniform amplitude scaling, uniform time scaling, uniform bi-scaling, time warping, and non-uniform amplitude scaling.

**Figure 3:**
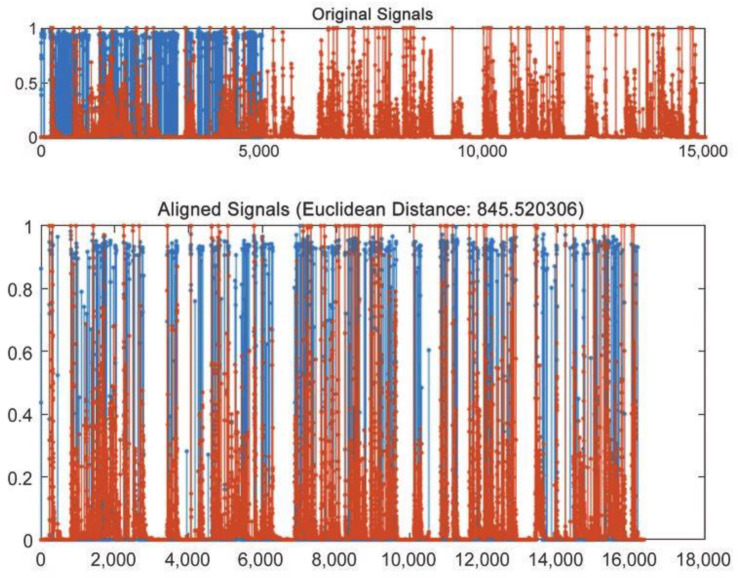
DTW result.

DTW has been proven a very effective similarity measure, since it minimizes the effects of shifting and distortion in time [60]. In this study, the sampling rate of current and voltage is different from that of spark image, and the data obtained by sampling is different in length. DTW algorithm is used for similarity, and the following results are obtained as Figure 3.

### 2.4. Spark Feature

The relationship between discharge pulse and discharge states is investigated by lots of previous research. The features of the spark image, which was provided previously, contain essential and significant information about processing parameters and conditions in WEDM such as current, power, wire direction, workpiece erosion. However, the spark in a spark image does not disappear immediately and its morphological and motion features also do not appear immediately. As a result, the relationship between spark image feature and discharge states is not directly and entirely related rather than non-linear and multi-frame corresponding.

#### 2.4.1. Sequence to Sequence Model

**Figure 4:**
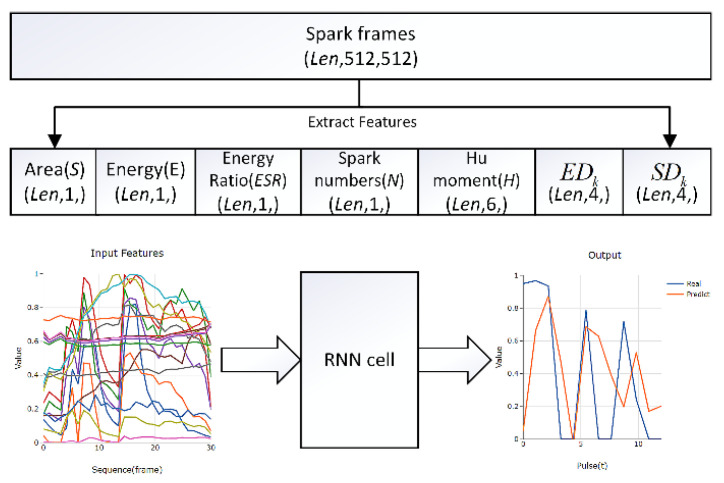
“Sequence to sequence” model.

According to the calculation method of the spark features, all of features extracted by the spark frames form into a feature array like (*Len*,18,1), where *Len* is the number of spark frames in a process. In Figure 4, the first model proposed in this paper is called as “Sequence to sequence model” which is based on RNN and takes the feature array of serval frames as input and the corresponding labels array as output. The output of RNN depends on several time step data. In other words, RNN can mine the relationship between frames in the spark image or its feature sequence due to the memory function of its network structure [61]. Hence, it can accurately predict the processing states through serval frames of spark images.

Given a sequence of inputs (*x*_1_, …, *x_T_*), a standard RNN computes a sequence of outputs (*y*_1_, …, *y_T_*) by iterating the equation

(22) $$h_{t}=\tanh(W^{hx}x_{t}+W^{hh}h_{t-1})$$

where *W^hx^* and *W^hh^* denote the weight of input layer and hidden layer of RNN, respectively.

*h_t−*1*_* denotes the output of hidden layer of RNN at the last time.

(23) $$y_{t}=W^{yh}h_{t}$$

where *W^yh^* denotes the weight of output layer and *h_t_* denotes the output of hidden layer of RNN at the present.

The traditional RNN is proved to have the problem of vanishing gradient [62].

**Figure 5:**
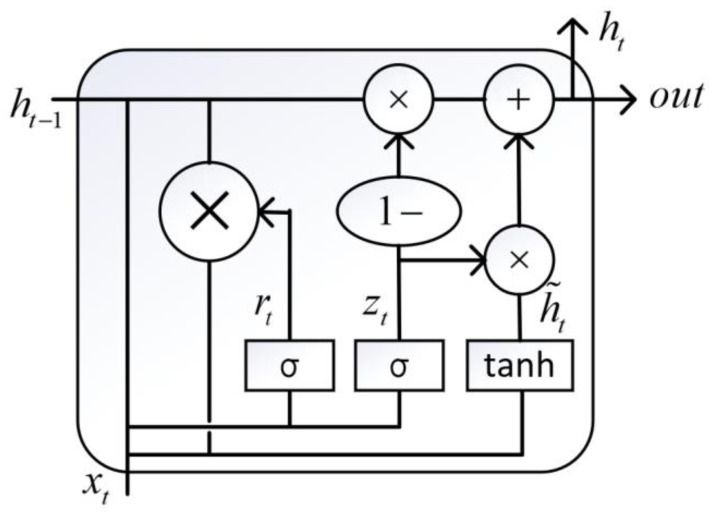
The structure of GRU cell.

Gated recurrent unit (GRU) is an improvement of traditional RNN which has the advantages of fewer parameters and learning about long-term dependence [63,64]. The struct of GRU is given by Figure 5. Update gate is used to decide whether to pass previous O/P (*h_t_*_−1_) to next cell (as *h_t_*) or not. Forget gate is nothing but additional mathematical operations with a new set of weights (*W_t_*). The variables in Figure 5 are updated by the following formula:

(24) $$z_{t}=\sigma (W_{z}\cdot [h_{t-1},x_{t}])$$

(25) $$r_{t}=\sigma (W_{r}\cdot [h_{t-1},x_{t}])$$

(26) $${h^{\prime }}_{t}=\tanh(W\cdot [r_{t}*h_{t-1},x_{t}])$$

(27) $$h_{t}=(1-z_{t})*h_{t-1}+z_{t}*{h^{\prime }}_{t}$$

where, *x_t_* is the input vector, *h_t_* is the output vector, *h^′^_t_* is candidate activation vector, *z_t_* is update gate vector, *r_t_* is reset gate vector, *W* is parameter, σ is a sigmoid activation function while *tanh* is a hyperbolic tangent activation function.

#### 2.4.2. Sequence to Sequence Model

**Figure 6:**
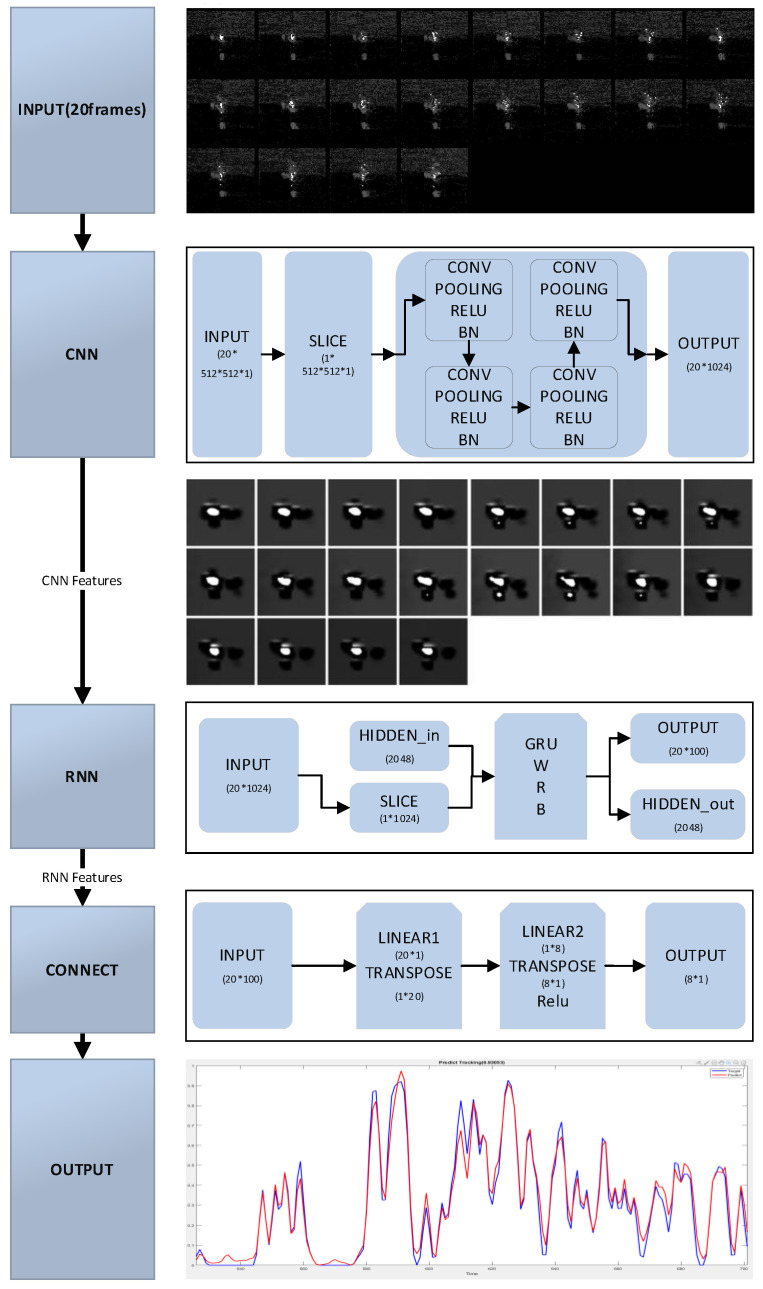
“Image to sequence” model.

In Figure 6, another model proposed in this article is called as “image to sequence” model. It is combined CNN network with RNN network.

Different from the above “sequence to sequence” model, the features were extracted by CNN, rather than extracted by the invariant calculation method provided at the first of paper. CNN is widely used to image feature extraction [65]. In Figure 6, the basic block of CNN contains convolutional layer, max pooling layer, *ReLU* active layer and batch normalization layer. Mathematically, the computational process can be described as

(28) $$Conv{\left(I,K\right)}_{x,y,c}=\sum_{i=1}^{n_{H}}\sum_{j=1}^{n_{W}}K_{i,j,c}I_{x+i-1,y+j-1,c}$$

(29) $$Maxpl{\left(I,K\right)}_{x,y,c}=\underset{m=0,\cdots ,K_{0}H-1}{\max}\underset{n=0,\cdots ,K_{1}W-1}{\max}(I_{h+m,w+n,c})$$

(30) ReLU(x)=max(0,x)

(31) $$BN(x)=\frac{x-E[x]}{\sqrt{Var[x]+\varepsilon }}*\gamma +\beta$$

where, *I* and *K* is the input image and kernel, and *x* is the input value of *ReLU* activation function or Batch normalization function. In *BN* operation, ε is added in the denominator for numerical stability and is arbitrarily small constant, and the parameters *γ* and *β* are subsequently learned in the optimization process.

Then, the output of CNN connects to RNN’s input in order to mining the relationship between each frames’ features. Because of difference in length between frames and discharge states calculated by current and voltage, a connect part is useful and necessary to match these two unequal sequences. That is, after inputting the RNN’s output to the connect part, the discharge states of WEDM are obtained by the finally output of the connect part.

To sum up, the “image to sequence” model extract spatial features (the features of one spark frame) through using CNN, and then the temporal features (the features of serval previous frames) are extracted by RNN.

Above two models would be trained by the samples of experiments. All of samples were separated into the train, validation and the test sets.

## 3. Data Synchronous Acquisition and Preprocessing

### 3.1. Synchronous Acquisition of Spark Image and Voltage Data

The spark image is captured by the High-Speed camera MEMRECAM ACS-1 M60 which is manufactured by *NAC Image Technology Inc.*, Tokyo, Japan. The voltage is measured by NI USB-6366 device which is manufactured by National Instruments Inc., Texas City, TX, USA. An acquisition and control for WEDM based on the LabVIEW tool is developed to synchronize the acquisition of image and voltage data.

**Figure 7:**
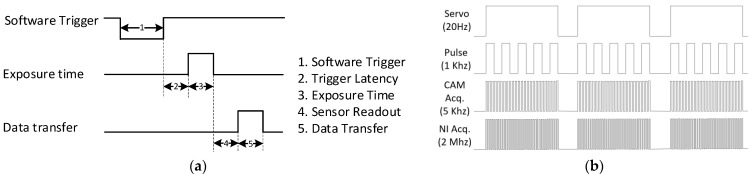
Software trigger timing: (**a**) camera timing; (**b**) acquisition and control timing.

According to the synchronous software of High-Speed camera, the time from a software trigger initiation to the start of shutter is shown below (as shown in Figure 7):

LabVIEW generates the signal to make NI device to acquire the voltage and output an external signal in order to control the High-Speed camera to capture a list of frames.

At the same time, LabVIEW control MCU device to output square wave signal to control WEDM movement such as generate pulse, servo movement, and so on.

**Figure 8:**
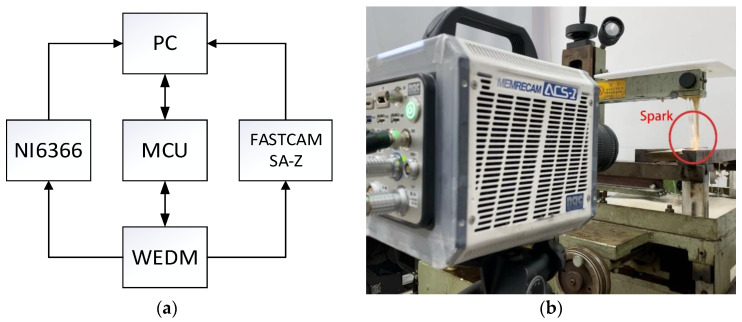
Experimental system: (**a**) control system; (**b**) experimental equipment.

After the acquisition and control system are established, the timing sequence of WEDM control and data synchronous acquisition is shown in Figure 8. Under the condition of no short circuit, the private server is given a constant value in the collection process. NI device generates synchronous clock signal to the High-Speed camera and then output trigger signal after waiting for the stable output of the clock signal.

### 3.2. Spark Feature

The waveform data was filtered by median value and the image data was filtered by background difference algorithm.

#### 3.2.1. Waveform Data

The waveform data was filtered by the method of median. In detail, let *r_l_* represents the filter left rank, and *r_r_* represents the filter right rank, then the result of xi the median *y_i_* of {*x_i_*−*r_l_*, …, *x_i_*−1, *x_i_*, *x_i_*+1, …, *xi+r_r_*} which is a subset of input sequence.

**Figure 9:**
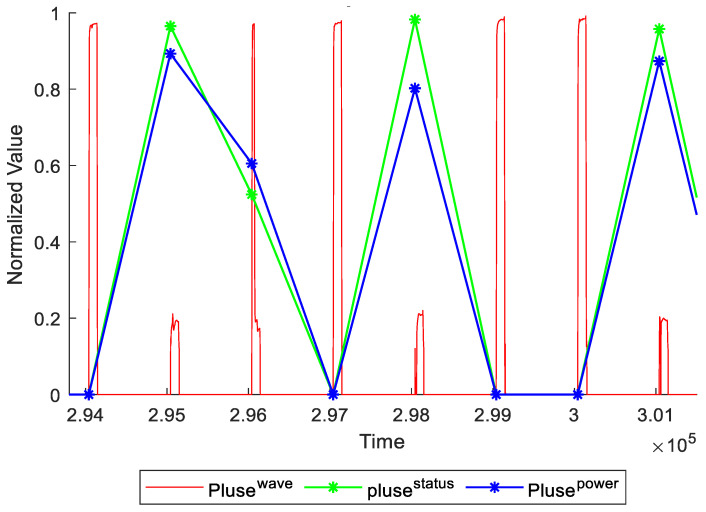
The label of pulse waveform.

In Figure 9, the red line represents to pulse waveform, while the green line represents the area of wave during current voltage pulse duration.

And the blue line represents to the power of wave corresponding to green line. The power is calculated as

(32) $$p=\sum_{t=r_{s}}^{r_{s}+t_{d}}voltage(t)\cdot current(t)$$

where *r_s_* is the rising edge time, and *t_d_* is the pulse duration.

#### 3.2.2. Image Data

The image can be expressed as

(33) Y=X+α+β

where *Y* is the output image, *X* is the real spark image, *α* represents the internal noise which includes camera noise and line noise, while *β* represents the external noise which includes environmental disturbances and background objects.

The spark has the property of high brightness because of the high energy in the discharge. Hence, in the process of acquiring image, the signal energy of the spark region is much larger than that of the noise. At the same time, during the cutting process, the background changes relatively little, and the ambient objects in the background under the condition of low exposure time set by the high-speed camera, the signal energy is negligible compared with the spark.

**Figure 10:**
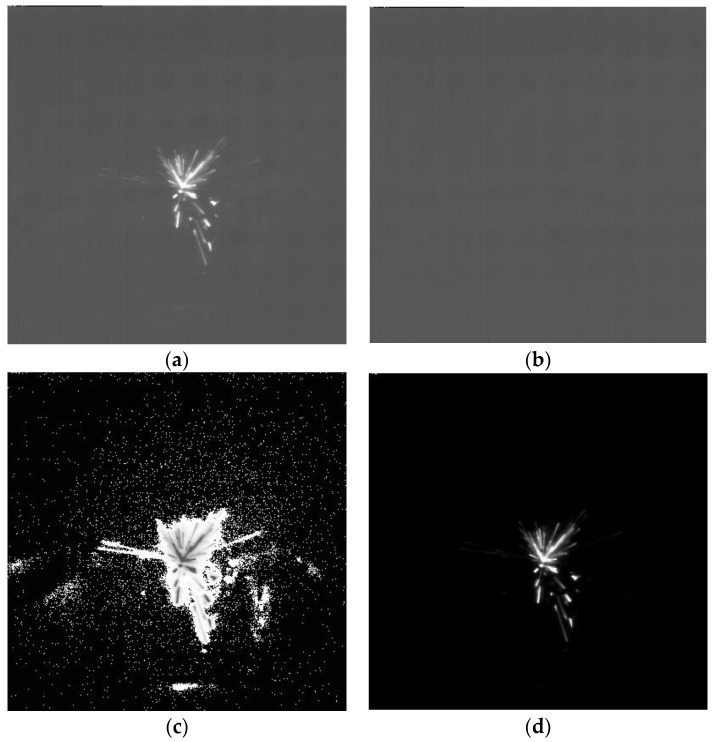
Image filter process (**a**–**d**): (**a**) original (*Y_t_*); (**b**) background (*Y*_0_); (**c**) difference (*Y_bd_*); (**d**) objective (X).

By background difference algorithm, the environmental disturbance and most of the noise with small change can be removed. As shown in Figure 10, the difference image (*Y_bd_*) is obtained by subtracting the background image (*Y*_0_) from the current frame (*Y_t_*).

(34) $$Y_{bd}=Y_{t}-Y_{0}$$

Then, the residual random noise is removed by pixel brightness threshold and pixel inversion.

(35) $$X=\{\begin{array}{cc} 0, & 0\leq Y_{bd}<k\\ Y_{bd}, & Y_{bd}\geq k \end{array}$$

**Figure 11:**
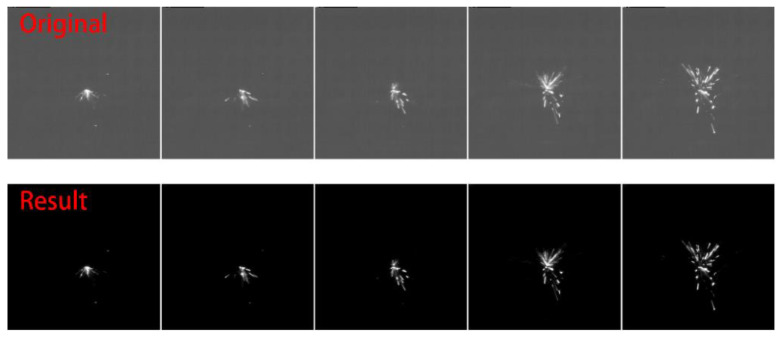
Filter result.

Finally, the filter results are shown in Figure 11.

## 4. Experiments and Analytics

A three-axis WEDM machine was used to conduct machining experiments. The work piece was AISI 1045 carbon steel (Table 2), which was widely used in industrial production. The other machining parameters in each experiment are shown in Table 3.

Under the condition of Nyquist, the pulse data acquisition frequency was set to 200 kHz while the image data acquisition frequency was set to 5000 fps (see Table 4). A total of 10,000 frames and 4,000,000 pulses were collected synchronously during the machining process.

The pulse frequency *f* is computed as

(36) $$f=\frac{1}{T}$$

where *T* is the pulse cycle.

If the pulse frequency is 5 kHz, it is calculated that the spark image data of 1 cycle corresponds to the pulse data of 1 cycle, that is 1 spark frame corresponds to 400 pulse points. The higher the image sampling rate, the more detail information is restored in a pulse cycle. Through the experiments of high-speed camera, we found that the sampling rate of 5 kHz is the best.

### 4.1. Analysis of Statistical of Experimental Data

**Figure 12:**
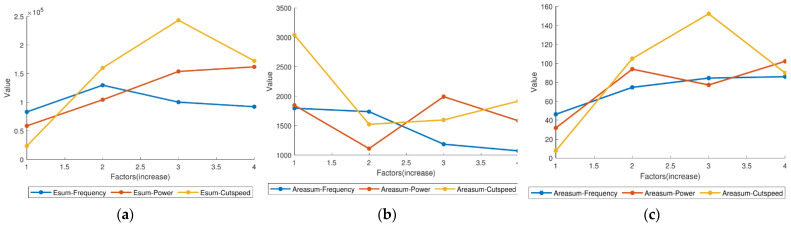
(**a**) Total energy of different factors; (**b**) total area of different factors; (**c**) total *ESR* of different factors.

Table 5 shows the statistical data of each experiment. For clearly showing, the curves about energy, area and *ESR* of different factors have plotted by Figure 12.

In Figure 12a, the total energy values of spark (*E_sum_*) are not much affected by pulse frequency due to the fact that the total energy of electricity is related to duty ratio but rarely related to frequency. The phenomenon of small fluctuation on the frequency curve are due to the different probabilities of spark occurring at different frequencies. Hence, *E_sum_* will fluctuate naturally. Additionally, it is speculated that under a certain processing frequency, *E_sum_* can reach the maximum. However, it cannot be directly proved the fact because there are not enough experiments on the influence of a single factor of frequency in this study. Affected by the power of processing pulse, *E_sum_* also changes greatly. Under the condition of maximum machining power, *E_sum_* is close to three times that of the original value. At the same time, *E_sum_* is greatly affected by the cutting speed and shows a trend of rising first and then falling, which indicates that within a certain range, increasing the cutting speed will increase *E_sum_*. Nevertheless, too high cutting speed will lead to partially short or even short circuit, and no spark will be generated, then *E_sum_* will also decrease.

Figure 12b shows the trend of the relationship between the total area values of spark (*S_sum_*) and each parameter. It can be seen that *S_sum_* is negatively correlated with the pulse frequency, that is, the higher the processing pulse frequency is, the smaller *S_sum_* is. However, the relationship between *S_sum_* and machining power or cutting speed is uncertain. The ratio of energy to area (*ESR*), which combines the relationship between *E_sum_* and *S_sum_*, focuses on reflecting the spark image information of the processing center, while ignoring the diffused spark image information. In this way, *ESR* can mostly directly reflects the processing state at the current moment. As shown in Figure 12c, *ESR* ends to increase with the increase of the processing pulse frequency, or the processing pulse power, or the cutting speed. This conclusion reflects that occurrence frequency and amplitude (brightness) of spark discharge occurring at the center point both increase with the increase of the three parameters mentioned above.

**Figure 13:**
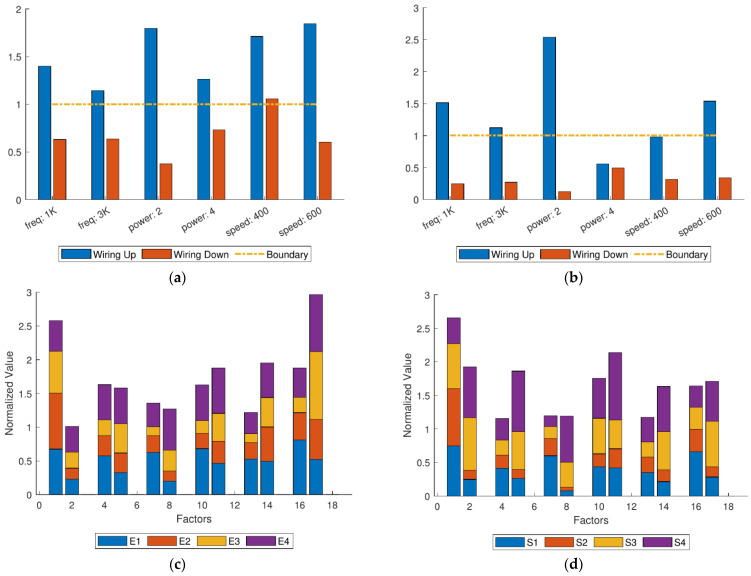
(**a**) *E_up_*:*E_down_* with different wire directions; (**b**) *S_up_*:*S_down_* with different wire directions; (**c**) energy distribution; (**d**) area distribution.

Furthermore, Figure 13a shows the ratio of the spark energy above and below the workpiece (*E_up_*:*E_down_*) where ‘above’ is the position 1 and position 2 of distribution, that is E1+E2. Similarly, Figure 13b shows the ratio of the spark area above and below the workpiece (*S_up_*:*S_down_*). The result showed that under the same circumstances of other processing conditions (except direction of wire), *E_up_*:*E_down_* and *S_up_*:*S_down_* are above 1 predominantly for the up direction of wire while less than 1 for the down direction of wire. To sum up, *E_up_* > *E_down_* and *S_up_* > *S_down_* are true. By observing the experimental phenomenon, when wire goes up, more sparks will explode on the top. This is mainly because the fragments of exploded spark will be affected by the force of the wire. In details, Figure 13c,d showed the distribution of the spark energy and area in each part under different wire direction. Consequently, the energy and area of spark are evenly distributed on the left (position 2 or position 3) and right (position 1 or position 4), no matter on the top or the bottom. To sum up, the direction of wire can affect the distribution of the up and bottom rather than the left and right.

**Figure 14:**
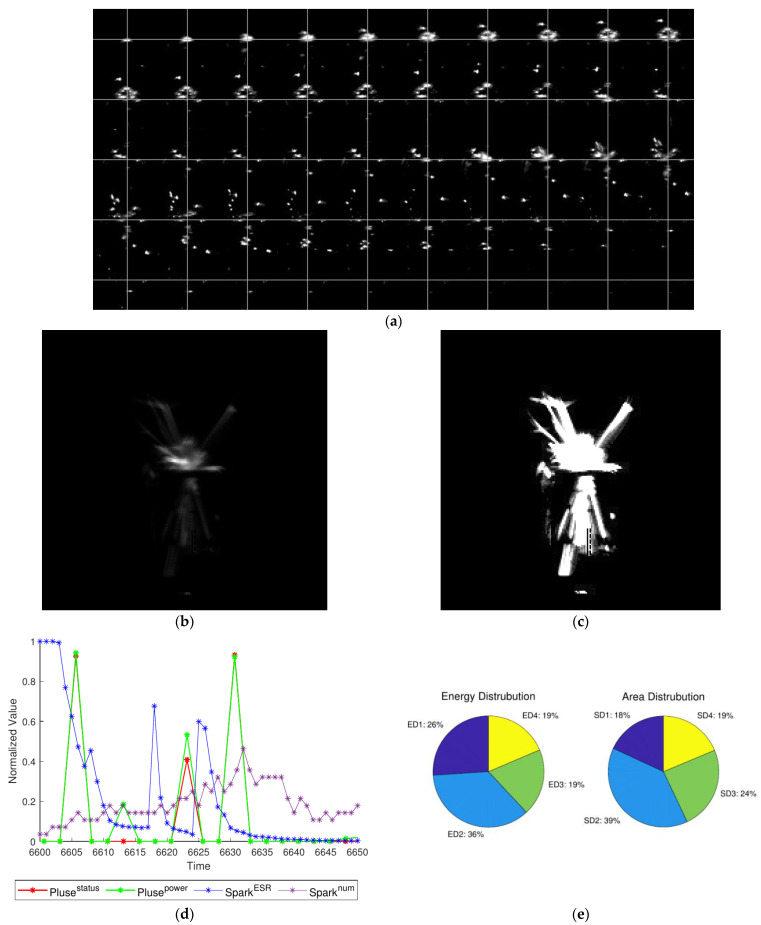
Single spark process (under the condition of trial 1): (**a**) image time series of machining process (50 frames); (**b**,**c**) combine image with normalization or binarization; (**d**) pulse waveform and spark features; (**e**) energy and area distribution.

**Figure 15:**
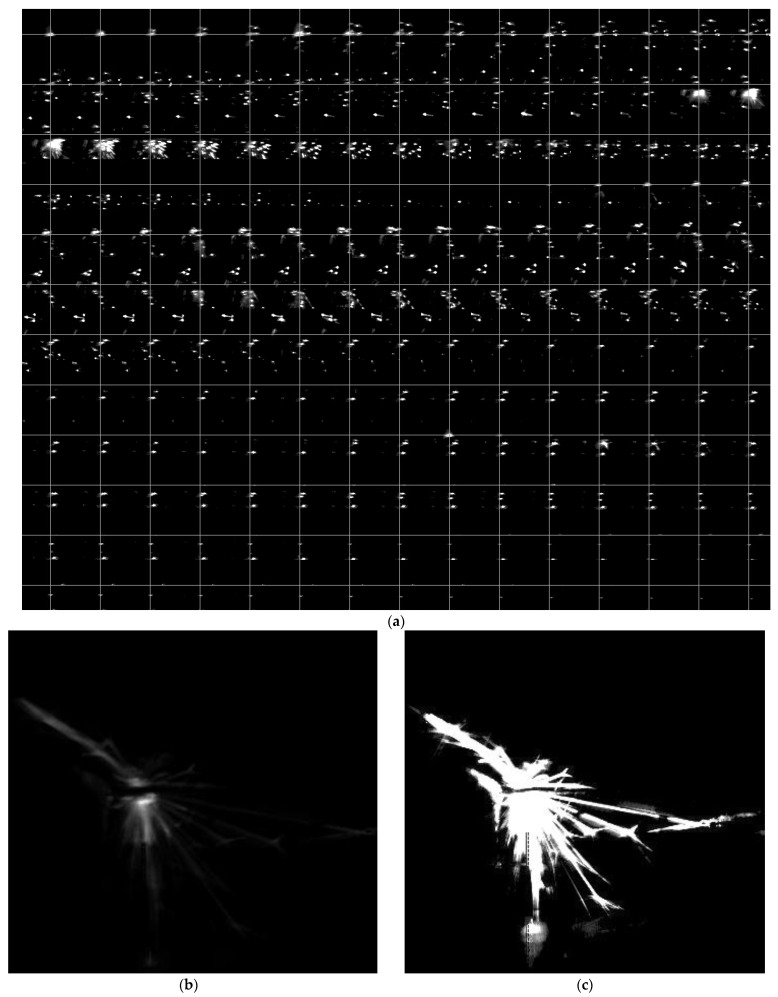

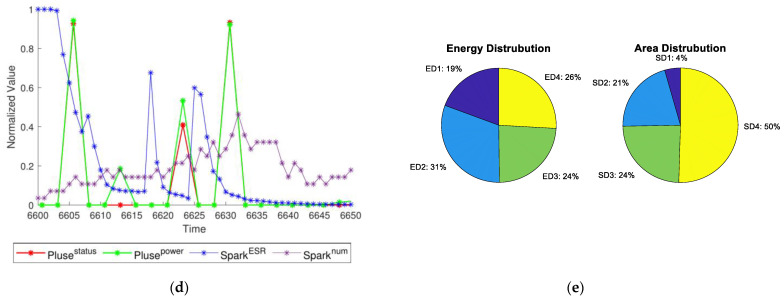
Multiple sparks process (under the condition of trial 1): (**a**) image time series of machining process (180 frames); (**b**,**c**) combine image with normalization or binarization; (**d**) pulse waveform and spark features; (**e**) energy and area distribution.

In order to visualize a spark process during experiment, Figure 14 and Figure 15 show the details of image and statistical data. Here describes the process about spark in Figure 14.

At normal, the pulse is on open states and the spark image shows none of spark which means all characteristic values are zero. When start to spark, the pulse changes into processing status and the spark image shows one small spark point which the shape is approximately a circle distributed in the processing center. It has a small area (*S_n_*) but a large amount of energy (*E_n_*) i.e., energy ratio (*ESR*) is very high. In the next frames, the number, area, energy, and average speed of spark show the characteristic of increasing and then decreasing. In a spark process (50 frames) (Figure 14), the *ESR* is the first feature to reach the peak because pulses of processing status are mainly in the previous period. The number of *ESR* peaks is correlated with the number of processing pulses (Figure 14c). Generally, the area of spark has a more stable trend because the spark that has occurred has a steady dissipating process. Its peak always lags behind the peak of energy and leads to a higher *ESR* when the spark occurs. This, in turn, supports the fact that the *ESR* peaked in the first place. In terms of feature distribution, the energy and area values of the four quadrants mainly are affected by the direction of wire. A composite image of 50 frames of spark images captured during the machining process is shown in Figure 14b. The left image in Figure 14b shows the brightness of spark while the right image shows the shape and distribution of spark. In details, the pie chart is shown in Figure 14d.

### 4.2. Training Results and Discussion

**Figure 16:**
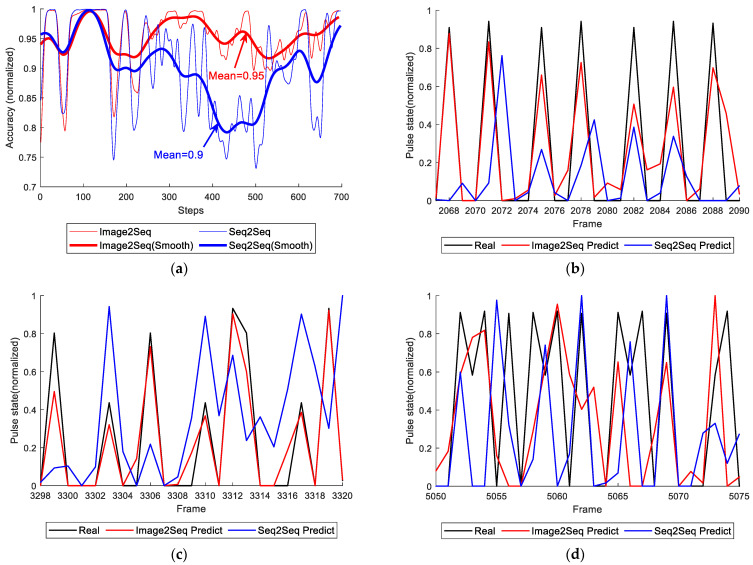
Predict accuracy and tracking result (under the condition of trial 1): (**a**) test accuracy; (**b**) prediction tracking 1; (**c**) prediction tracking 2; (**d**) prediction tracking 3.

**Figure 17:**
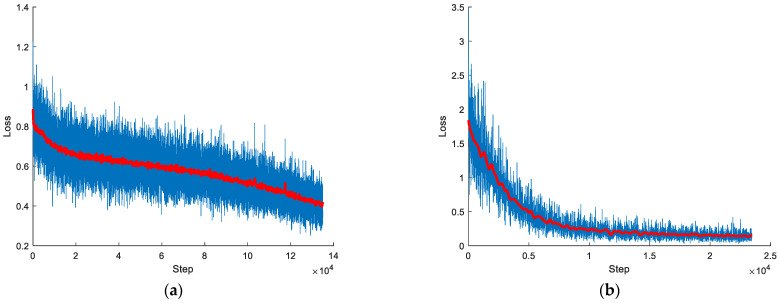
Train result (under the condition of trial 1): (**a**) loss of “sequence to sequence” model; (**b**) loss of “image to sequence” model.

Figure 16 and Figure 17 show the predict result and train result of both models, respectively. In Figure 16a, it is found that RNN could predict discharge states to some extent, but the result was unsatisfactory. No matter how to adjust the length of input or the layers of RNN, the result could not improve at all. The training loss of “sequence to sequence” model is shown in Figure 17a. It is obvious that the loss is unstable though it has a trend to descend. The model occurred this result due to the input dataset which has the main features, but they are not enough to restore the ordinary spark image data. Hence, the advantage of the model is fast training while the disadvantage is that the precision of prediction is not high. Figure 17b is the loss of “image to sequence” model. It shows some vibration and a gradual downward trend. Figure 16b,d show the tracking of prediction. The dataset of testing includes the training dataset (frame 3000 to 15,000) and testing dataset (frame 1 to 3000). The predict tracking of training dataset showed in Figure 16c works well. It is clearly found that it can tail after the label. Meanwhile, the number of peak and the value of peak nearly equal to the label. When it turns to the testing dataset, it also remained the result of peak number equaling.

Compare to the “sequence to sequence” model, the “image to sequence” model extracts the features automatically. To sum up, the “image to sequence” model has slow training speed but high prediction accuracy while the “sequence to sequence” model trains the model fast accompany with low prediction accuracy. The mean of precision of the whole dataset is 95% in “image to sequence” model, while it is 90% in “sequence to sequence” model.

## 5. Conclusions

The motivation of this paper is to analyze the spark image of wire electrical discharge machining using image processing and machine learning technology. First, the relationship between spark images and discharge status is studied by image feature extraction through traditional algorithms. It is concluded that the spark image features are related to the discharge status. To predict the discharge status by spark image, a CNN-GRU is proposed, which extracts the image feature by CNN and predict the discharge status by GRU. Experimental results show that the proposed model performs better comparing to the GRU model. The contributions of this paper are as follows:

Firstly, different from the traditional research perspective, this paper proposes a new perspective to study the machining state of WEDM, that is, to predict the machining state by WEDM image. It is found that during the process of machining, the pulse waveform of “short” status may not represent the status of non-machining. Hence, it is difficult to recognize the “short” and “short discharge” by the electrical parameters. However, the spark image can provide obvious evidence to recognize them. Because the spark images have the certain morphological and kinematic characteristics, and they are direct phenomena of the machining process so that they can represent the status. Additionally, the above spark images’ characteristics are more regular than pulse waveform’s characteristics. By using traditional image feature extraction method, the regularities between image and spark are obtained through experimental analysis. They are summarized as follows:

In the machining process, the power of the discharge pulse directly affects the sum of the spark energy (*E_sum_*) calculated from the image. Meanwhile, within a certain range, the higher the cutting speed is, the greater the *E_sum_* will be. However, too fast cutting speed will lead to short circuit and the *E_sum_* will decrease consequently.

The spark area of the image (*S_sum_*) is negatively correlated with the discharge frequency, and its relationship with the discharge power and cutting speed is not correlated. The energy density (*ESR*) of spark image focuses on the machining center points, so it can directly reflect the processing state. Within a certain range of cutting speed, its value is positively correlated with discharge frequency, power, and cutting speed. The spark distributions (includes area and energy) of the image are mainly related to the wire direction.

Secondly, this paper is among the first to present an approach to define the discharge status by using continuous quantity. The advantages of this approach are: (1) it improves the stability; (2) ease in converging the model. It is beneficial to design the deep learning model to explore the relationship between discharge status and spark images.

Thirdly, the proposed model named “sequence to sequence” was used to explore the relationship between spark characteristics and discharge status. Further, the proposed model named “image to sequence” was trained to extract the features of spark image by CNN and identify the discharge status by GRU. Experimental results show that spark images can accurately predict and track the machining status. The precision of the whole dataset is 95% in “image to sequence” model and is 90% in “sequence to sequence” model.

In this paper, the regularities between the spark image and the discharge state are studied by the statistical analysis and deep learning model. In the future work, the “image to sequence” model and method presented in this paper can be further improved in the aspect of accuracy, stability and speed. Future research directions could be conducted as follows: on-line monitoring discharge state and closed-loop control system of WEDM based spark images. This paper also provides a spark image-based solution for monitoring the discharge state of multi-groove WEDM.

## Figures and Tables

**Table 1:** Features and representation information.

Features	Representation Information
Area	Represents the area of the spark in the image. To some extent, it reflects the amount of erosion in processing.
Energy	Represents the energy of the spark in the image. It is closely related to processing parameters such as current and voltage.
Energy density	Reflects the concentration of energy. It is the amount of energy per unit area which is closely related to the processing state of processing center.
Spark area distribution	Represents the area distribution of processing region. It is closely related to wire direction
Spark energy distribution	Represents the Energy distribution of processing region. It is closely related to wire direction.
Spark number	Represents the numbers of the spark. It reflects the morphological characteristics of spark process, such as the gathering spark generated by the discharge and the dissipating spark generated by the open circuit.
HU moment	Represents other geometric features of the spark region in the image which are invariant to rotation, translation, scale, and so on.

**Table 2:** Workpiece properties.

Workpiece Properties	Value
Carbon, C	0.43–0.50%
Density	7.87 g/cm^3^
Hardness Thermal conductivity	163 HB 51.9 W/mK

**Table 3:** Experiment conditions.

Trials	Control Parameters	Frequency (kHz)	Power (Level)	Cutting Speed (step/s)	Wire Direction	Purpose
1	Compared	2	3	500	Down	Find the best cam fps and shutter time
2	Frequency	1	3	500	Down	Change frequency, occur open, normal, arc, short
3		3	3	500	Down	
4		4	3	500	Down	
5		5	3	500	Down	
6	Power	2	1	500	Down	Change power, occur open, normal, arc, short
7		2	2	500	Down	
8		2	4	500	Down	
9		2	6	500	Down	
10	Cutting Speed	2	3	200	Down	Change speed, occur open, normal, arc, short
11		2	3	300	Down	
12		2	3	400	Down	
13		2	3	500	Down	
14		2	3	600	Down	
15	Pump Direction	2	3	500	Up	Change pump direction, occur open, normal, arc, short
16		1	3	500	Up	
17		3	3	500	Up	
18		2	2	500	Up	
19		2	4	500	Up	
20		2	3	400	Up	
21		2	3	600	Up	

**Table 4:** Acquisition conditions.

Acquisition Conditions	Value
Pulse sample frequency	2,000,000 Hz
Image sample frequency	5000 fps
Workpiece	AISI 1045 carbon steel

**Table 5:** Statistical data.

Trials	Energy Distribution				Area Distribution				$\frac{E_{1}+E_{2}}{E_{3}+E_{4}}$
1	38005.3	31755.8	33175.8	45947.9	558.0	385.5	256.6	534.8	0.805
2	18827.9	13348.1	19769.9	31254.4	233.9	124.1	734.3	705.6	0.631
3	26852.4	23716.8	35663.6	43684.5	246.6	122.9	529.5	840.6	0.637
4	20804.6	16697.8	25513.1	37261.5	162.0	121.4	375.3	528.6	0.597
5	20312.6	14901.6	20677.1	36413.5	145.3	64.6	319.3	545.0	0.617
6	4616.5	5378.5	19099.4	29740.8	47.6	19.3	616.1	1164.9	0.205
7	16415.4	12204.9	25470.1	50335.0	73.1	48.0	353.5	637.6	0.378
8	38046.9	27019.3	33910.0	54941.8	390.3	268.8	401.5	933.6	0.732
9	43115.0	33755.8	35175.8	49947.9	258.0	279.4	422.9	625.3	0.903
10	2210.0	1445.6	10002.9	11150.4	283.3	247.5	1406.0	973.9	0.173
11	4132.5	3245.6	7429.4	8865.6	548.4	356.3	1109.3	1026.5	0.453
12	40590.1	41708.5	36084.5	41746.6	200.8	165.4	533.1	625.4	1.057
13	42960.7	48778.7	82123.4	69625.1	265.3	141.6	637.4	554.3	0.605
14	58808.4	61857.2	26016.4	26288.1	324.7	231.1	666.9	702.2	2.307
15	58435.9	84406.6	109940.7	59632.9	348.2	381.3	790.3	442.6	0.842
16	55418.4	68259.8	50711.6	37577.6	698.4	796.0	626.9	360.4	1.401
17	47125.8	24401.0	19724.4	42737.0	386.0	185.5	206.1	302.5	1.145
18	51452.0	20110.3	10978.6	28841.8	562.0	240.5	164.9	151.4	1.797
19	55871.2	18679.2	15657.0	433663.1	409.3	179.1	495.0	558.4	1.263
20	43375.0	19750.0	10767.5	26125.0	326.3	216.3	211.3	342.5	1.711
21	66363.6	33506.5	18701.3	35454.5	619.5	309.1	309.1	294.8	1.844

## Nomenclature

*W*	Width of the spark image
*H*	Height and width of the spark image
*q_w_*	Heat flux of spark
*q*_0_	Maximum heat flux *q*_0_ of spark
*R_pc_*	Equivalent heat input radius
*F_c_*	Fraction of total EDM spark power
*V*	Discharge voltage (V)
*I*	Discharge current (A)
*S*	Spark area (units)
*S_up_*	Spark area above the workpiece (units)
*S_down_*	Spark area below the workpiece (units)
*S_sum_*	Total area values of spark images (units)
*E*	Spark energy (units)
*E_up_*	Spark energy above the workpiece (units)
*E_down_*	Spark energy below the workpiece (units)
*E_sum_*	Total energy values (units)
*ESR*	Spark energy density (-)
*SD_k_*	Spark area distribution (units)
*ED_k_*	Spark energy distribution (units)
*m_pq_*	Classical geometric moments of an image
*I_xy_*	Pixel value of spark image
*SR*	Surface roughness (µm)
*SV*	Spark gap voltage (V)
*T_on_*	Pulse on time (µs)
*T_off_*	Pulse off time (µs)
*WF*	Wire-speed feed (m/s)
*MRR*	Material removal rate (mm/s)
*TWR*	Tool electrode wear rate (-)
*SR*	Surface roughness (µm)
*tanh*	Hyperbolic tangent activation function
*Conv*	Convolutional layer
*Maxpl*	Max pooling layer
*ReLU*	*ReLU* active layer
*BN*	Batch normalization layer
WEDM	Wire electrical discharge machining
EDM	Electro/Electrical discharge machining
WEDT	Wire electrical discharge turning
CNN	Convolution neural network
RNN	Recurrent neural network
GRU	Gated recurrent unit
LSTM	Long short-term memory
DTW	Dynamic time warping
FEM	Finite element modeling
WEDT	Wire electrical discharge turning
GRP	Gaussian process regression
MOGA	Multi-objective genetic algorithm
ANN	Artificial neural network
ANFIS	Adaptive neuro-fuzzy inference system
ARAS	Additive ratio assessment
AHP	Analytical hierarchy process
BPNN	Neural network with back propagation algorithm
GA	Genetic algorithm
MSE	Mean-squared error
LWPA	Strategy of the leader
USV	Ultrasonic vibration
MF	Magnetic field
WMA	Wavelet moment analysis
HMA	Hu moment analysis
FDA	Fractal dimension analysis
GC	Local geometric characteristics
GGC	Global geometric characteristics
SVM	Support vector machine
ANOVA	Analysis of variance
NI	National Instruments

## References

[1] Kavimani V., Prakash K.S., Thankachan T. (2019). Influence of machining parameters on wire electrical discharge machining performance of reduced graphene oxide/magnesium composite and its surface integrity characteristics. Compos. Part B Eng..

[2] Schaller P.R., Hollenstein D.C., Rappaz P.M., Wälder D.G., Winter P.J. (2006). Characterization of electrical discharge machining plasmas.

[3] Shabgard M., Ahmadi R., Seyedzavvar M., Oliaei S.N.B. (2013). Mathematical and numerical modeling of the effect of input-parameters on the flushing efficiency of plasma channel in EDM process. Int. J. Mach. Tools Manuf..

[4] Ho K.H., Newman S.T., Rahimifard S., Allen R.D. (2004). State of the art in wire electrical discharge machining (WEDM). Int. J. Mach. Tools Manuf..

[5] Ahmed N., Naeem M.A., Rehman A.U., Rafaqat M., Umer U., Ragab A.E. (2020). High Aspect Ratio Thin-Walled Structures in D2 Steel through Wire Electric Discharge Machining (EDM). Micromachines.

[6] Saleh M., Anwar S., El-Tamimi A., Khan Mohammed M., Ahmad S. (2020). Milling Microchannels in Monel 400 Alloy by Wire EDM: An Experimental Analysis. Micromachines.

[7] Ho K.H., Newman S.T. (2003). State of the art electrical discharge machining (EDM). Int. J. Mach. Tools Manuf..

[8] Shankar P., Jain V.K., Sundararajan T. (1997). Analysis of Spark Profiles during Edm Process. Mach. Sci. Technol..

[9] Ablyaz T.R., Muratov K.R. (2016). The technological quality control of stack cutting by wire electrical discharge machining. Surf. Rev. Lett..

[10] Dekeyser W., Snoeys R., Jennes M. (1988). Expert system for wire cutting EDM, based on pulse classification and thermal modeling. Robot. Comput. Integr. Manuf..

[11] Gostimirovic M., Kovac P., Sekulic M., Skoric B. (2012). Influence of discharge energy on machining characteristics in EDM. J. Mech. Sci. Technol..

[12] Joshi S.N., Pande S.S. (2011). Intelligent process modeling and optimization of die-sinking electric discharge machining. Appl. Soft Comput..

[13] Giridharan A., Samuel G.L. (2014). Modeling and analysis of crater formation during wire electrical discharge turning (WEDT) process. Int. J. Adv. Manuf. Technol..

[14] Assarzadeh S., Ghoreishi M. A neural network approach for powder mixed electrical discharge machining (PMEDM) modeling and optimization. Proceedings of the Ninth Cairo University International Conference on Mechanical Design and Production.

[15] Chaudhari R., Vora J.J., Mani Prabu S.S., Palani I.A., Patel V.K., Parikh D.M., de Lacalle L.N.L. (2019). Multi-Response Optimization of WEDM Process Parameters for Machining of Superelastic Nitinol Shape-Memory Alloy Using a Heat-Transfer Search Algorithm. Materials.

[16] Yuan J., Wang K., Yu T., Fang M. (2008). Reliable multi-objective optimization of high-speed WEDM process based on Gaussian process regression. Int. J. Mach. Tools Manuf..

[17] Sivasankar S., Jeyapaul R. (2016). Modelling of an Artificial Neural Network for Electrical Discharge Machining of Hot Pressed Zirconium Diboride-Silicon Carbide Composites. Trans. Famena.

[18] Moghaddam M.A., Kolahan F. (2015). An optimised back propagation neural network approach and simulated annealing algorithm towards optimisation of EDM process parameters. Int. J. Manuf. Res..

[19] Spedding T.A., Wang Z.Q. (1997). Parametric optimization and surface characterization of wire electrical discharge machining process. Precis. Eng..

[20] Markopoulos A.P., Manolakos D.E., Vaxevanidis N.M. (2008). Artificial neural network models for the prediction of surface roughness in electrical discharge machining. J. Intell. Manuf..

[21] Liao Y.S., Yan M.T., Chang C.C. (2000). A neural network approach for the on-line estimation of workpiece height in WEDM. J. Mater. Process. Technol..

[22] Naresh C., Bose P.S.C., Rao C.S.P. (2020). Artificial neural networks and adaptive neuro-fuzzy models for predicting WEDM machining responses of Nitinol alloy: Comparative study. SN Appl. Sci..

[23] Sidhu S.S., Batish A., Kumar S. (2013). Neural network–based modeling to predict residual stresses during electric discharge machining of Al/SiC metal matrix composites. Proc. Inst. Mech. Eng. Part B J. Eng. Manuf..

[24] Upadhyay A., Prakash V., Sharma V. (2018). Optimizing Material Removal Rate Using Artificial Neural Network for Micro-EDM. Design and Optimization of Mechanical Engineering Products.

[25] Sagbas A., Kahraman F., Esme U. (2012). Optimization of Wire Electrical Discharge Machining Process Using Taguchi Method and Back Propagation Neural Network. Eskişehir Osman. Üniversitesi Mühendislik Mimar. Fakültesi Derg..

[26] Shakeri S., Ghassemi A., Hassani M., Hajian A. (2015). Investigation of material removal rate and surface roughness in wire electrical discharge machining process for cementation alloy steel using artificial neural network. Int. J. Adv. Manuf. Technol..

[27] Sen B., Hussain S.A.I., Gupta A.D., Gupta M.K., Pimenov D.Y., Mikołajczyk T. (2020). Application of Type-2 Fuzzy AHP-ARAS for Selecting Optimal WEDM Parameters. Metals.

[28] Suganthi X.H., Natarajan U., Sathiyamurthy S., Chidambaram K. (2013). Prediction of quality responses in micro-EDM process using an adaptive neuro-fuzzy inference system (ANFIS) model. Int. J. Adv. Manuf. Technol..

[29] Sarkheyli A., Zain A.M., Sharif S. (2013). A multi-performance prediction model based on ANFIS and new modified-GA for machining processes. J. Intell. Manuf..

[30] Somashekhar K.P., Ramachandran N., Mathew J. (2010). Optimization of Material Removal Rate in Micro-EDM Using Artificial Neural Network and Genetic Algorithms. Mater. Manuf. Process..

[31] Ong P., Chong C.H., Rahim M.Z.b., Lee W.K., Sia C.K., Ahmad M.A.H.B. (2018). Intelligent approach for process modelling and optimization on electrical discharge machining of polycrystalline diamond. J. Intell. Manuf..

[32] Ming W., Hou J., Zhang Z., Huang H., Xu Z., Zhang G., Huang Y. (2015). Integrated ANN-LWPA for cutting parameter optimization in WEDM. Int. J. Adv. Manuf. Technol..

[33] Yan M.T., Liao Y.S., Chang C.C. (2001). On-line Estimation of Workpiece Height by Using Neural Networks and Hierarchical Adaptive Control of WEDM. Int. J. Adv. Manuf. Technol..

[34] Yan M.T., Li H.P., Liang J.F. (1999). The Application of Fuzzy Control Strategy in Servo Feed Control of Wire Electrical Discharge Machining. Int. J. Adv. Manuf. Technol..

[35] Zhang Z., Huang H., Ming W., Xu Z., Huang Y., Zhang G. (2016). Study on machining characteristics of WEDM with ultrasonic vibration and magnetic fifield assisted techniques. J. Mater. Process. Technol..

[36] Chen Z., Zhang Y., Zhang G., Huang Y., Liu C. (2017). Theoretical and experimental study of magnetic-assisted finish cutting ferromagnetic material in WEDM. Int. J. Mach. Tools Manuf..

[37] Ablyaz T.R., Bains P.S., Sidhu S.S., Muratov K.R., Shlykov E.S. (2021). Impact of Magnetic Field Environment on the EDM Performance of Al-SiC Metal Matrix Composite. Micromachines.

[38] Marrocco V., Modica F., Bellantone V., Medri V., Fassi I. (2020). Pulse-Type Influence on the Micro-EDM Milling Machinability of Si3N4-TiN Workpieces. Micromachines.

[39] Aggarwal V., Pruncu C.I., Singh J., Sharma S., Pimenov D.Y. (2020). Empirical Investigations during WEDM of Ni-27Cu-3.15Al-2Fe-1.5Mn Based Superalloy for High Temperature Corrosion Resistance Applications. Materials.

[40] Gurupavan H.R., Ravindra H.V., Devegowda T.M., Addamani R. (2018). Machine Vision System for Correlating Wire Electrode Status and Machined Surface in WEDM of AlSi3N4 MMC’S. IOP Conf. Ser. Mater. Sci. Eng..

[41] Sanchez J.A., Plaza S., De Lacalle L.N.L., Lamikiz A. (2006). Computer simulation of wire-EDM taper-cutting. Int. J. Comput. Integr. Manuf..

[42] Liu Z., Chen H., Pan H., Qiu M., Tian Z. (2014). Automatic control of WEDM servo for silicon processing using current pulse probability detection. Int. J. Adv. Manuf. Technol..

[43] Zhang Z., Ming W., Zhang G., Huang Y., Wen X., Huang H. (2015). A new method for on-line monitoring discharge pulse in WEDM-MS process. Int. J. Adv. Manuf. Technol..

[44] Yang Y., Yanghan M., Tian H. Research of the micro-EDM discharge state detection method based on matlab Fuzzy control. Proceedings of the 2010 International Conference on Mechanic Automation and Control Engineering.

[45] Liu W., Jia Z., Zou S., Zhang L. (2014). A real-time predictive control method of discharge state for micro-EDM based on calamities grey prediction theory. Int. J. Adv. Manuf. Technol..

[46] Maity K., Mishra H. (2016). ANN modelling and Elitist teaching learning approach for multi-objective optimization of μ-EDM. J. Intell. Manuf..

[47] Zhao Z., Li Y., Liu C., Gao J. (2019). On-line part deformation prediction based on deep learning. J. Intell. Manuf..

[48] Tseng K.H., Chang C.Y., Cahyadi Y., Chung M.Y., Hsieh C.L. (2020). Development of Proportional-Integrative-Derivative (PID) Optimized for the MicroElectric Discharge Machine Fabrication of Nano-Bismuth Colloid. Micromachines.

[49] Albawi S., Mohammed T.A. Understanding of a convolutional neural network. Proceedings of the 2017 International Conference on Engineering and Technology (ICET).

[50] Zhang X., Liu Y., Wu X., Niu Z. (2019). Intelligent pulse analysis of high-speed electrical discharge machining using different RNNs. J. Intell. Manuf..

[51] Lee K.B., Kim C.O. (2018). Recurrent feature-incorporated convolutional neural network for virtual metrology of the chemical mechanical planarization process. J. Intell. Manuf..

[52] Bustillo A., Urbikain G., Perez J.M., Pereira O.M., de Lacalle L.N.L. (2018). Smart optimization of a friction-drilling process based on boosting ensembles. J. Manuf. Syst..

[53] Chen X., Zhang B., Gao D. (2020). Bearing fault diagnosis base on multi-scale CNN and LSTM model. J. Intell. Manuf..

[54] Zhang F., Gu L., Zhao W. Study of the Gaussian Distribution of Heat Flux for Micro-EDM. Proceedings of the ASME 2015 International Manufacturing Science and Engineering Conference.

[55] Ikai T., Fujita I., Hashiguchi K. (1992). Heat input radius for crater formation in the electric discharge machining. EEJ Trans. Ind. Appl..

[56] Hu M.-K. (1962). Visual pattern recognition by moment invariants. IRE Trans. Inf. Theory.

[57] Žunić J., Hirota K., Rosin P.L. (2010). A Hu moment invariant as a shape circularity measure. Pattern Recognit..

[58] Wu Z., Jiang S., Zhou X., Wang Y., Zuo Y., Wu Z., Liang L., Liu Q. (2020). Application of image retrieval based on convolutional neural networks and Hu invariant moment algorithm in computer telecommunications. Comput. Commun..

[59] Cassisi C., Montalto P., Aliotta M., Cannata A., Pulvirenti A. (2012). Similarity Measures and Dimensionality Reduction Techniques for Time Series Data Mining. Advances in Data Mining Knowledge Discovery and Applications.

[60] Shou Y., Mamoulis N., Cheung D.W. (2005). Fast and ExactWarping of Time Series Using Adaptive Segmental Approximations. Dep. Comput. Sci..

[61] Mou L., Ghamisi P., Zhu X.X. (2017). Deep Recurrent Neural Networks for Hyperspectral Image Classification. IEEE Trans. Geosci. Remote. Sens..

[62] Pearlmutter B.A. (1995). Gradient Calculations for Dynamic Recurrent Neural Networks: A Survey. IEEE Trans. Neural Netw..

[63] Wang Y., Liu M., Bao Z., Zhang S. (2018). Short-Term Load Forecasting with Multi-Source Data Using Gated Recurrent Unit Neural Networks. Energies.

[64] Dey R., Salem F.M. Gate-variants of gated recurrent unit (GRU) neural networks. Proceedings of the IEEE 60th international midwest symposium on circuits and systems (MWSCAS).

[65] Hinton G.E., Salakhutdinov R.R. (2006). Reducing the dimensionality of data with neural networks. Science.

